# Research and TLS (LiDAR) Construction Diagnostics of Clay Brick Masonry Arched Stairs

**DOI:** 10.3390/ma15020552

**Published:** 2022-01-12

**Authors:** Rafał Nowak, Tomasz Kania, Radosław Rutkowski, Ewa Ekiert

**Affiliations:** 1 Department of General Civil Engineering, Faculty of Civil and Environmental Engineering, West Pomeranian University of Technology in Szczecin, Piastów Ave. 50a, 70-311 Szczecin, Poland; 2 Department of General Civil Engineering, Faculty of Civil Engineering, Wrocław University of Science and Technology, Wybrzeże Wyspiańskiego 27, 50-370 Wrocław, Poland; tomasz.kania@pwr.edu.pl; 3 Department of Technological Processes, Faculty of Economics and Transport Engineering, Maritime University of Szczecin, Wały Chrobrego 1-2, 70-500 Szczecin, Poland; szczecin.ar@gmail.com; 4 Department of Chemical Inorganic Technology and Environment Engineering, Faculty of Chemical Technology and Engineering, West Pomeranian University of Technology in Szczecin, Piastów Ave. 42, 71-065 Szczecin, Poland; ewa.dabrowa@zut.edu.pl

**Keywords:** stairs, masonry, clay brick, arch, vault, management, quality, light detection and ranging (LiDAR), TLS

## Abstract

The study presents the terrestrial laser scanning (TLS) diagnostic of the clay brick masonry arched staircase in a historic building. Based on the measurements of the existing arched stair flights, 1:1 scale experimental models with and without stair treads were made. Strength tests of the models were carried out for different concentrated force locations in relation to the supporting structure. Force, deflections and reaction in the upper support of the run were measured during the tests. The influence of the masonry steps on the curved vault on the load capacity and stiffness of the run structure was analyzed. The conducted experimental investigations showed that the key element responsible for the actual load-bearing capacity and stiffness of this type of stair flights were the treads above the masonry arch.

## 1. Introduction

**Figure 1:**
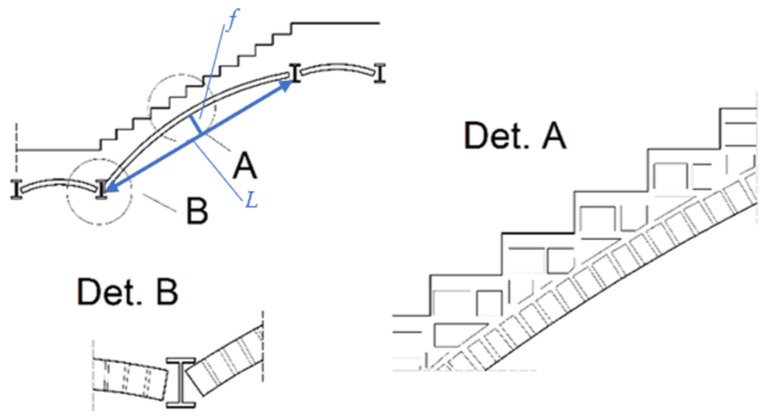
Masonry stairs with segmental vault and arched staircase slab, based on [1].

**Figure 2:**
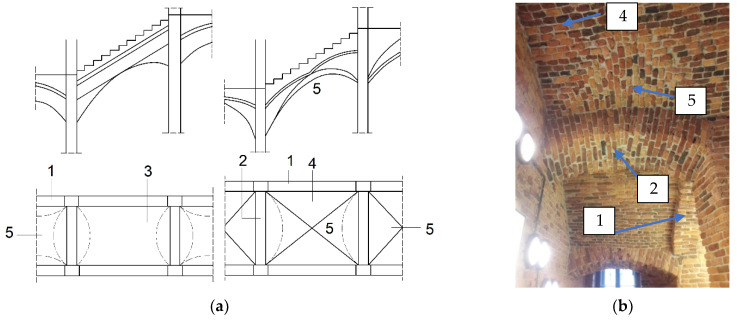
Brick stairs with column supports based on [1]. Scheme (**a**) and partial photo (**b**): 1—arched masonry stair sides; 2—arched masonry bolt; 3—barrel staircase with support on sides; 4—groin vault in stair flight; 5—groin or barrel vaults in stair landing.

In the authors’ countries, in historical buildings from the 19th century, masonry arch stairs were commonly used to connect floors [1]. The arches were shaped between steel beams as a load-bearing span for the staircase landings and flights. At that time, the technology of reinforced concrete was not yet known, so steel and masonry construction was the most popular, especially in residential buildings. The construction of staircases was mostly in the form of segmental or cross vaults. The structure of the flights was made as a stair arch with the thickness of half a brick (12 cm) and elevation *f* = 1/12–1/14 *L* [1] (Figure 1 and Figure 2).

A flight of arch stairs is characterized by a slender, long structure with a slope of approximately 35°–40°. One of the main permanent loads on the flight of stairs are the bricks along the entire flight. The steps have the least bricks in the middle of the stairs’ arch and the most at the ends. The steps are not connected with a typical masonry bond. The bricks must be cut to fit the actual geometry of the arches.

The flights and landings were supported by steel beams. The construction of flights had a good fire resistance (except for the steel beams), stiffness and durability. The constructions of brick stairs are still in use despite many years of service. Additionally, they are a testament of old times and often protected by the conservator as a part of cultural heritage. Their condition often raises concern because of the damages, mainly cracks or erosion of joints. The typical damages of brick staircase construction are: degradation of mortar and bricks, flattening of vaults and cracking. The damages arise as a result of overloading, wrongly executed repair works and dynamic loads. Sometimes, one can find constructional errors such as improper fixing of gear plates on I-beam flanges.

**Figure 3:**
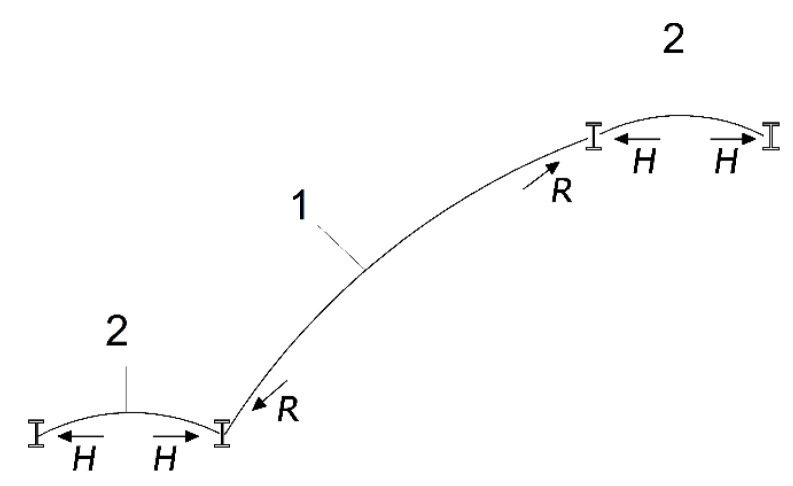
Calculation scheme of masonry stairs with segmental vault and arched staircase slab: 1—arched staircase slab; 2—landings.

Information on calculation methods or principles of construction is often difficult to find. This type of construction was created mainly on the basis of experience with brick vaults. The steel beams supporting the vaults were calculated by the methods available at that time [1]. The operation of landing vaults is similar to balcony or roof vaults. On the other hand, staircase slabs are at an angle depending on their length and height. The distribution of stresses and internal forces is different from other staircase structures. A calculation scheme of an example of an arched masonry stairs with vaulted landings supported on steel beams is shown in Figure 3.

**Figure 4:**
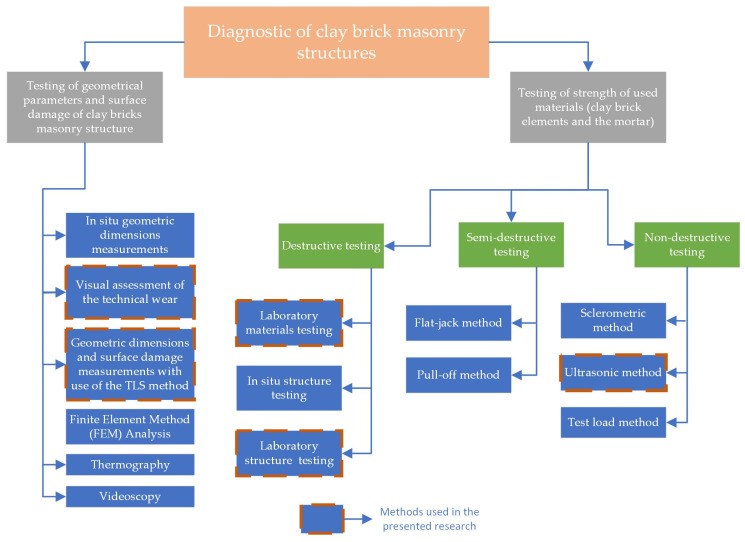
Flowchart for clay brick masonry structure diagnostics.

One of the most prevalent issues in this type of stairs is the necessity of diagnostics of their technical condition in order to confirm their durability and functional use. The chosen methods used in the diagnostics of masonry structures in the authors’ countries are shown in Figure 4.

The diagnosis of masonry structures primarily focuses on a visual assessment in order to detect cracks and damages [2,3,4,5]. The early diagnostics usually uses non-destructive measuring equipment, which allows the preliminary characteristics of existing materials or the degree of their degradation to be evaluated [6,7,8,9,10]. Those methods most often include sclerometric measurements (Schmidt hammer) or ultrasonic measurements. At that stage of diagnosis, infrared thermography is a novel and useful method for historic plaster and painted vaults. The management of the data derived from the application of infrared thermography, integrated with the information from visual inspections, the architectural survey and the historic analysis, allows a complete characterization of the historic plasters and painted masonry vaults to be obtained [11].

A more accurate determination of material properties in masonry structures involves sampling. In the case of brick alone, this process is feasible; however, sampling of mortar is significantly more difficult. Therefore, in many diagnostic operations, core samples are taken, also containing a fragment of mortar and masonry elements adjacent on both sides. The core samples, after certain correlations, are used to determine the load-bearing capacity of the whole masonry structure [12,13,14,15,16,17]. Even more accurate results may be obtained by test-loading a masonry section with actuators [18]. However, this method requires making cuts in the masonry in order to locate the beams to place the actuators. The method is mostly used as a validation is structural elements that are to undergo alteration, repair, or reconstruction.

A similar effect can be obtained if a section of the masonry is cut out and transported to the laboratory for experimental testing [14,18]. A less destructive solution is the flat-jack method [18,19]. It requires appropriate calibration procedures and precise preparation of the measurement base. Additionally, it may be imprecise in low buildings and lead to permanent damage when testing masonry with weak lime mortar [18]. The best measurement method is to perform a loading test with appropriate measurement [20,21,22,23,24], which does not lead to the destruction of the structure but allows the correlation between stresses and strains to be obtained, in order to assess the actual load-bearing capacity and stiffness. Final results on the load-bearing capacity of the structure can only be obtained from experimental destructive testing.

The finite element method (FEM) is a fundamental analysis for the assessment of masonry in seismic zones [25,26,27,28]. Nonlinear static calculation methods are commonly adopted for the evaluation of seismic performance [26,27,28,29,30,31,32]. Laurenco et al. [28] presented a research study on that subject with a yield criterion that included different strengths along each material axis. Baraldi et al. [31] presented the rigid beam model for studying the dynamic behavior of cantilever unreinforced masonry walls, considered along their thickness and subjected to out-of-plane loading. Celano et al. [32] presented research on the in-plane resistance of masonry walls with the use of two modeling approaches, a finite element model and a discrete macro-element model, with the use of non-linear analyses.

The TLS (terrestrial laser scanning) measurements using 3D scanners are starting to be used for structural diagnosis. Three-dimensional LiDAR (light detection and ranging) scanners have revolutionized the capability and accuracy of geometry measurements in construction, shipbuilding and other areas of science and technology [33,34,35,36,37,38,39,40,41,42,43,44,45,46,47,48,49]. TLS can be used to measure damage to the surface layers of materials, including bricks and mortar, as well as to track moisture in structures [33,41,42,43,44,45,46,47,48,49]. TLS in structural analysis is primarily used to measure strain and deflections, as well as deformations, of structural elements [25,42,43,44,45,46,47,48,49,50]. This technique can also be used for control measurements at the stage of constructing [51]. Some researchers use TLS technology to monitor the condition of construction to prevent possible damage [52,53]. Additionally, detailed geometry studies using TLS are useful in post-disaster analyses of structures, including earthquakes [53].

The assessment of the technical condition of brick stairs is not an easy task. The first step usually consists of initial observations of the geometry and of looking for visible signs of damage. Usually, the construction is protected by plaster, so some defects may be hidden. Modern TLS measuring methods can also be helpful in preliminary surveys. Only by uncovering, it is possible to fully assess the quality of brick and mortar and their degree of degradation. The evaluation of the material properties of the structure is possible by taking representative samples and performing laboratory tests [2,10,54,55,56,57,58,59]. A deeper analysis of the performance of the structure and the causes of its damage is possible using FEM-based 3D models [57,58,59,60,61,62,63,64]. On this basis, it is possible to precisely select the reinforcement methods. The analysis in the elastic range is rather easy to perform, but the analysis in the plastic range, after cracking has occurred, requires time-consuming and expensive studies [60].

Studies of masonry arches and vaults can be found in the works [61,62,63,64,65,66,67,68,69,70,71,72,73,74,75,76,77,78,79,80,81,82,83,84,85,86,87,88,89,90,91,92,93,94,95,96,97,98,99,100,101,102,103,104,105]. In these studies, masonry vaults are characterized by significant arch rise and placement of the masonry stairs. A small number of researchers has taken up the problem of load-bearing capacity of masonry stairs. Research has mostly focused on establishing the principles and methods for structural calculations [92,93,94,95,96,97,98,99]. There is a visible lack of research on masonry staircases. The design of staircases is similar to masonry arch work; however, the arrangement of loads is different. In addition, the influence of the steps above the vault on the operation of the system is also significant.

The article presents a novel approach, in which experimental tests for staircases were made in a 1:1 scale resembling a real-life construction. Together with laboratory tests, the TLS diagnosis of an arched staircase in a real-life historic building is presented. The history of the TLS LiDAR measurement method dates back only 20 years [41,42,106]. The authors’ research study indicates that it can be used in the diagnosis of some types of building structures [41,42]. It is important to assess the applicability of the TLS method in the diagnosis of masonry stair structures, including arched staircases. The possibilities of its use in this area are presented in this article.

## 2. Real-Life Structure

### 2.1. Case Study Description

**Figure 5:**
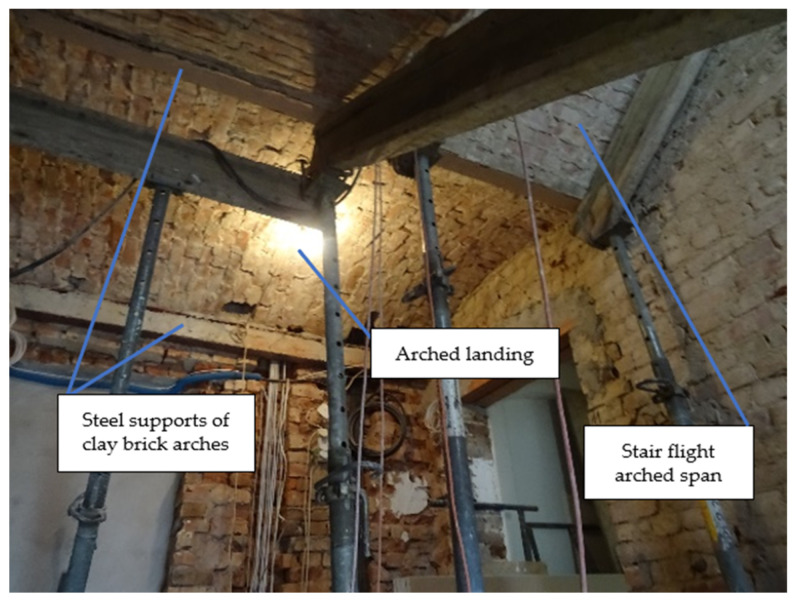
View of the researched masonry staircase.

The building had four residential floors and an attic. The ceilings on the upper floors were made of wooden beams, while, on the first floor, they were made as arched segmental vaults on steel beams. The floors were 3.2 m high. The staircase was 2.5 m wide and 5.3 m long. The length of the landings was 1.6–1.7 m and the length of the flights was 2.6 m, with a width of 1.2 m. The analyzed stair flights were at an angle of 38°. The stairs were made of steel and masonry, with arched spans between the landings. The rise of the stair arch had a value of *f* = 1/19 *L*. Figure 5 shows the general view of the arched stair structure under consideration.

The measured dimensions of the bricks were 25 × 12 × 6.5 cm^3^. The brick and mortar compressive strength was estimated with use of the non-destructive, ultrasonic measurements according to the dependencies described in [107]. The dependency of bricks is shown in Equation (1).

(1) $$f_{c,brick}=1.4949\cdot e^{0.002\;\cdot UPV}\;\;\left(\mathrm{MPa}\right),$$

where *f_c_*_,*brick*_ (MPa) is the compressive strength of the brick element and UPV (m/s) is the measured ultrasonic pulse velocity. The dependency of lime–cement mortar is presented in Equation (2).

(2) $$f_{c,mortar}=-5.5+0.007671\cdot UPV\;\;\left(\mathrm{MPa}\right),$$

where *f_c_*_,*mortar*_ (MPa) is the compressive strength of lime–cement mortar. The results of the tests are presented in Table 1.

The compressive strength of the bricks varied in a wide range, from 10.4 to 28.4 MPa with a mean value of 17.6 MPa. The compressive strength of lime–cement mortar varied from 2.1 to 5.4 MPa with a mean value of 4.1 MPa. Due to the large discrepancy found between the strength values of the masonry units and mortar, it was decided to carry out laboratory tests using materials with standard properties. In this respect, a brick class of 25 MPa and a mortar class of 5 MPa were assumed.

### 2.2. TLS Diagnostic on a Real-Life Structure

Stair geometry measurements and diagnostics were performed using TLS technology, using a stationary scanner Focus M70 (Faro, Lake Mary, FL, USA) with a single measurement accuracy of 0.2 mm. From the measurements, a point cloud in Autodesk Recap (RCP) format was obtained, which was then verified in CAD software (Version2021, San Rafael, Autodesk, CA, USA). RCP format files store spatially indexed point cloud data that can be processed in applications to view, edit and analyze object geometry. The purpose of the measurements was to analyze the geometry of the masonry arch. The aim of the study was to indicate the geometric irregularities, whose technical condition was visually inspected. After removing the plaster in the selected irregularity areas, the condition of masonry and mortar was evaluated.

**Figure 6:**
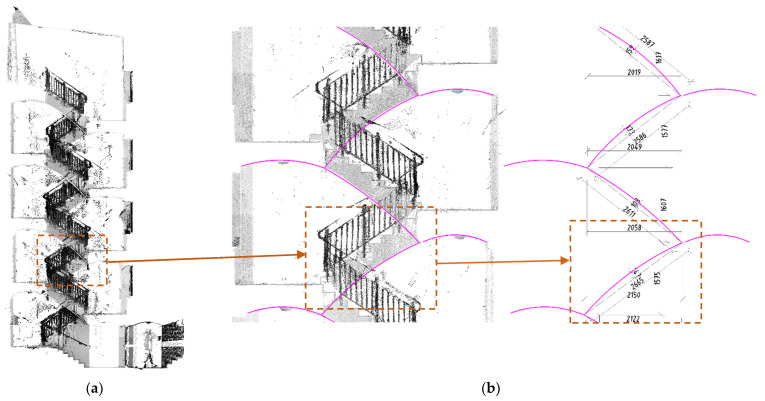
TLS data acquired from existing building: section view of building (**a**) and geometry measurements for analysis (**b**). The dashed line marks the stair flight which was subjected to further laboratory testing.

The TLS measurements allowed us to determine the exact geometry of the stair flight. Figure 6 shows a general view of the obtained TLS measurement map along with a picture of the geometry of the curved staircase of the building.

**Figure 7:**
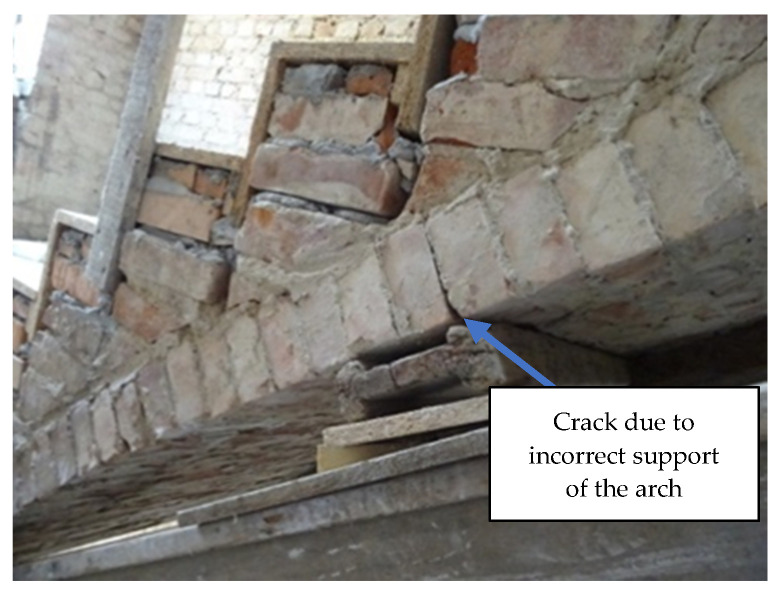
Section of the staircase with visible crack of the arch caused by improper renovation and deterioration of masonry joints.

Alongside TLS measurements, a visual evaluation of the mortar joints in the stair flights as well as of the treads was performed. Numerous damages and cracks were found. The most important was a crack in the joint on the masonry arch presented in Figure 7.

**Figure 8:**
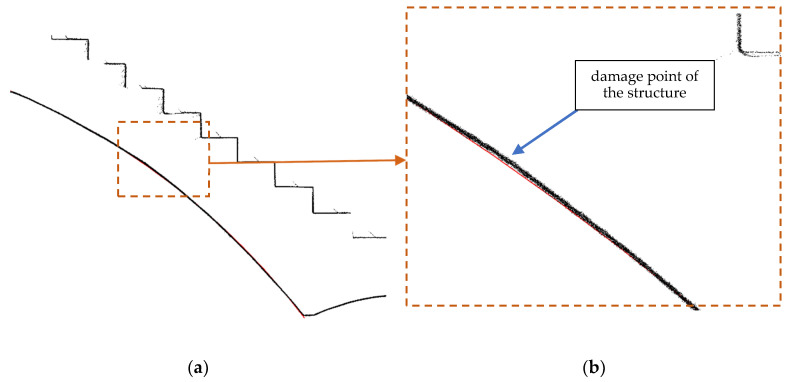
TLS data output for the point damage of the stair flight: section view of the stair flight (**a**) and point of damage (**b**).

Incorrectly made support changed the distribution of internal forces in the masonry arch, which was the reason for the whole structural diagnosis. Support should have been put in place for the entire surface of the arch in the form of a centering with an appropriate shape to match the actual geometry of each stair flight arch. Figure 8 shows the output of TLS diagnostics for the run fault of the stair flight.

The weakest points of the analyzed masonry arch structure were the joints between bricks and mortar. On the basis of the research work carried out, it was decided to reinforce the whole structure of flights and landings with a steel structure fixed from the bottom.

## 3. Laboratory Tests

### 3.1. Materials

The tested samples and elements presented in this research study were built using clay brick, class 25. The dimensions of the bricks were 25 × 12 × 6.5 cm^3^ (the same as in the real-life stair flight construction). For the preparation of joints, lime–cement mortar, class M5, was used. For the preparation of masonry mortar, a factory-made, dry, lime–cement mortar class M5 mixture was used (Quick-mix TWM-M5; Sievert, Strzelin, Poland). The mixture consisted of a cement binder, slaked lime, quartz fillers and refining additives. The thickness of the joint was about 1 cm. To determine the properties of the materials used, initial tests in accordance with current standards [108,109,110,111] were conducted. The results of the tests are presented in Table 2.

### 3.2. Laboratory Models of Stair Flights

**Figure 9:**
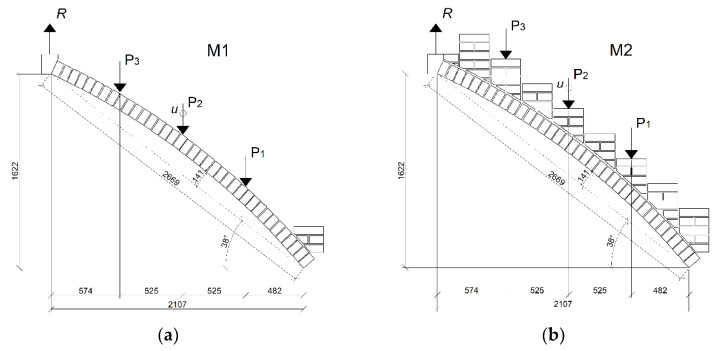
Laboratory models of stair flights (dimensions in mm): model M1—without treads (**a**); model M2—with treads (**b**). P_1_–P_3_—force application points; R—force from the arch registered with a force gauge; u—vertical displacement in the middle of the arch span.

Laboratory tests were conducted on two models of the stair flights—without stair treads (M1) and with stair treads (M2). The models were made in a 1:1 scale based on the measurements of the analyzed construction, with a width of a single 25 cm brick (Figure 9).

The dimensions of the flight were taken from the real-life structure and were noted as follows:

- Height 162 cm;
- Length 210 cm;
- Slope 38°;
- Arch rise *f* = 14 cm.

The stair flight construction was supported on a self-made test bench frame. During the masonry works, a wooden structure with expanded polystyrene was used as a centering for the arch. The support was removed while the arch structure was drying, so that the structure could be pressed down naturally. Due to indoor conditions, the bricks were soaked in water before construction, then wetted with water daily during the mortar curing processes. In order to take measurements, a force gauge was placed in the upper corner of the flight to transfer the vertical force.

Measurements were conducted with inductive sensors (for vertical displacements) and two force gauges connected to an MGC Plus HBM Hottinger Bridge. In addition, the bridge was connected to the ARAMIS optical three-dimensional displacement and strain measurement system. The model was painted on the back side with a white–black pattern to enable image correlation. First, the model was tested in the elastic range with a concentrated force applied at three different locations, P_1_, P_2_ and P_3_. At the force application locations, horizontal surfaces were prepared with quick-setting mortar with a strength, after 24 h, of at least 25 MPa. The assumed limit loads at points P_1_ and P_3_ were up to 1.5 kN, while the limit for point P_2_ was noted when the first crack was registered, after which the test was stopped. The loads at P_1_, P_2_ and P_3_ were applied separately to the tested specimen. Then, on the basis of the M1 model, the M2 model was created by adding treads over the staircase (Figure 3). In order to create the geometry of the steps, it was necessary to precisely cut the bricks at the angles correlating with the arch.

The model with treads (M2) was tested similarly to the model without treads (M1). Points P_1_, P_2_ and P_3_ were determined at the same locations as in the case of the M1 model. Assumed force limits for points P_1_ and P_3_ were up to 6 kN. At the middle point, P_2_, the structure was loaded up to failure. The loads were applied separately to the specimen.

### 3.3. Results of Laboratory Tests

The model without treads (M1) was loaded with concentrated forces P_1_ = P_3_ = 1.5 kN. The value of the force at point P_2_ was increased until the first crack occurred at the load value of 4.5 kN. This moment was considered to be the end of elastic work of the structure. Failure was caused by the opening of the crack within the joint located under the concentrated force at point P_2_.

**Figure 10:**
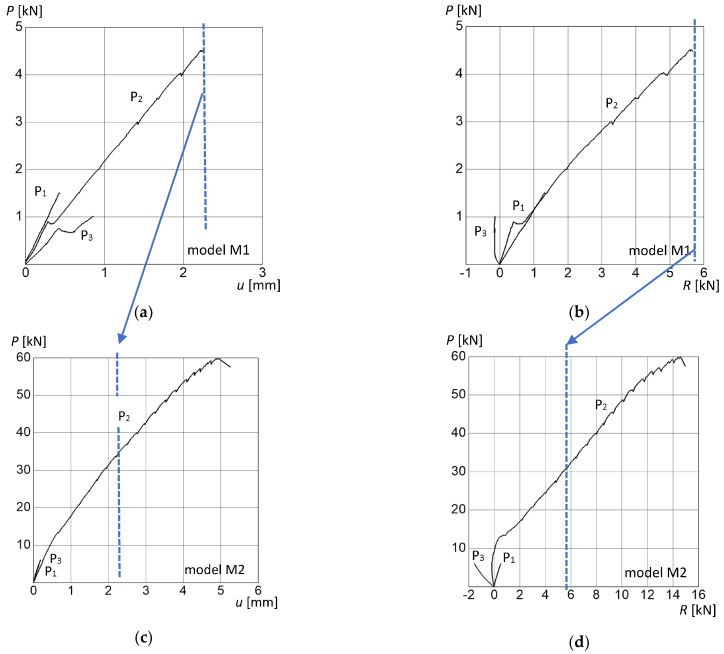
Experimental results of masonry staircases: relation P–u (vertical displacement in the middle of an arch in point P_2_) for model M1 (**a**); force P–reaction R for model M1 (**b**); force P–displacement u (vertical displacement in the middle of an arch in point P_2_) for model M2 (**c**); force P–reaction R for model M2 (**d**).

The model with masonry treads (M2) was loaded at the same locations as model M1 by applying concentrated forces on the masonry treads. At points P_1_ and P_3_, a structural load of P_1_ = P_3_ = 6 kN was applied. At the middle point, P_2_, the value of the load was increased until failure due to cracking, which occurred at a force of P = 59.8 kN. The results of the measurements of the displacement and support reactions of the researched models are presented in Figure 10.

**Figure 11:**
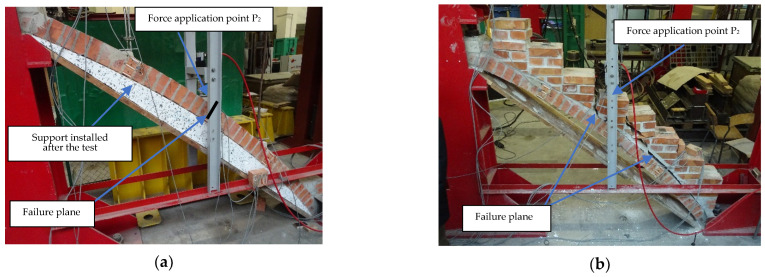

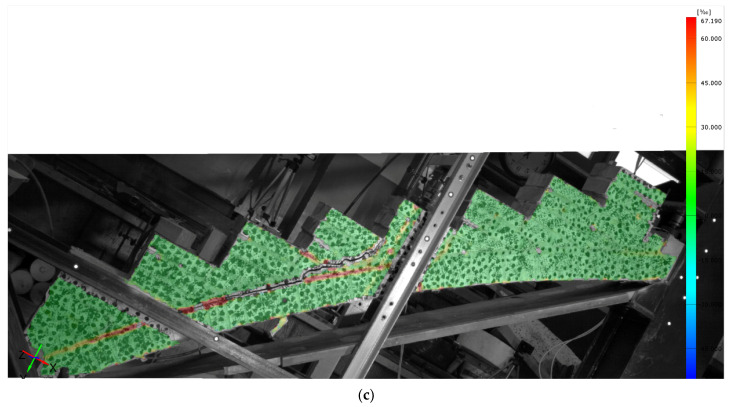
Registered failure mode of tested models of stair arches: model M1 (**a**), model M2 (**b**) and measurements from ARAMIS of deformation during failure of model M2 (**c**).

By including the treads in the stair flight curve analysis, the load capacity was considerably higher than previously assumed. The load-bearing capacity for the model with treads was 13.3 times higher than in the model without treads. The deflection for the same load level P = 4 kN at point P_2_ for model M1 was u = 1.9 mm and, for model M2, u = 0.2 mm. The deflection value of model M2 was 9.5 times smaller than the deflection of model M1. The steps masoned above the staircase significantly increased the load-bearing capacity of the structure, as well as its stiffness. Figure 11 shows the failure mode of the tested models of stair arches and the results of the measurement of the deformations for M2 made with the ARAMIS system at the moment of destruction.

The model of structural failure changed, which, in the case of model M2, occurred as a result of cracking along the arch, at the interface of the arch with the brick treads. First of all, the masonry above the arch detached from the rest of the structure (masoned brick treads). This proved the significant importance of the stair treads above the arch in its load-bearing capacity. Once the crack formed between the arch and the treads, the rest of the structure exhibited rapid failure.

For model M1 (without treads), the deflection measured vertically in the load range up to 0.9 kN developed similarly for points P_1_ and P_2_. The deflection curve for P_3_ was significantly different from that for P_1_ and P_2_. Comparing the curves for a load level P = 1 kN, it was found that the smallest deflection of the structure was recorded for the load at point P_1_ (48.2 cm from the lower support), for which a vertical deflection of the structure u = 0.3 mm was measured. At point P_2_ (100.7 cm from the lower support), the vertical deflection u = 0.4 mm was obtained. At point P_3_ (153.2 cm from the lower support and 57.4 cm from the upper support), the highest vertical deflection of the structure u = 0.8 mm was achieved. At point P_2_, the deflection was 1.3 times higher in relation to point P_1_, while, at point P_3_, it was 2.7 times higher than at P_1_. That is, the lower the location of force along the staircase was, the lower the influence on the deformation of the structure.

Changing the location of the force had also a significant effect on the recorded reaction R in the upper part of the staircase. For model M1, in the case of force applied to the middle and lower parts of the staircase (P_1_ and P_2_), the reaction was positive, which means that it was directed vertically downwards and balanced the horizontal forces in the arch. However, for the force at point P_3_, the situation was completely different. Due to the different geometry of the arch compared to typical vaults, the reaction had the opposite direction. The arch in this place did not generate compressive force on the upper support, but, rather, a tension. Comparing the values of the reaction R between different force points at the force level P = 1 kN, it should be noted that the reaction for points P_1_ and P_2_ was similar and was about R = 0.8 kN. For point P_3_, the reaction was R = −0.1 kN, which was eight times lower. The reactions for points P_1_ and P_2_ had different values until the inflection point on the P_2_ curve, at force P = 0.9 kN.

Different results were obtained for model M2 (with treads). The differences in deflection u were less visible between P_1_ and P_3_. For the load level P = 5 kN at points P_1_ and P_3_, the deflection had a value of u = 0.2 mm. In the investigated load range, the addition of stair treads increased the stiffness of the structure regardless of force location.

For model M2, the reaction forces in the support differed from each other. Negative values for the support were registered again for point P_3_ but also partially for point P_2_. The reaction in the case of the force at the center (P_2_) operated in a tension range, up to P = 9 kN, after which it started to work in compression. At the force level P = 5 kN, the reaction at point P_1_ was R = 0.4 kN; at point P_2_, it was R = −0.2 kN; and, at point P_3_, it was R = −1.4 kN. The spreading force for the force P = 5 kN was recorded only for the force at point P_1_. In the case of force at points P_2_ and P_3_, tension was observed. The value of the reaction for P_3_ was seven times higher than for P_2_.

**Figure 12:**
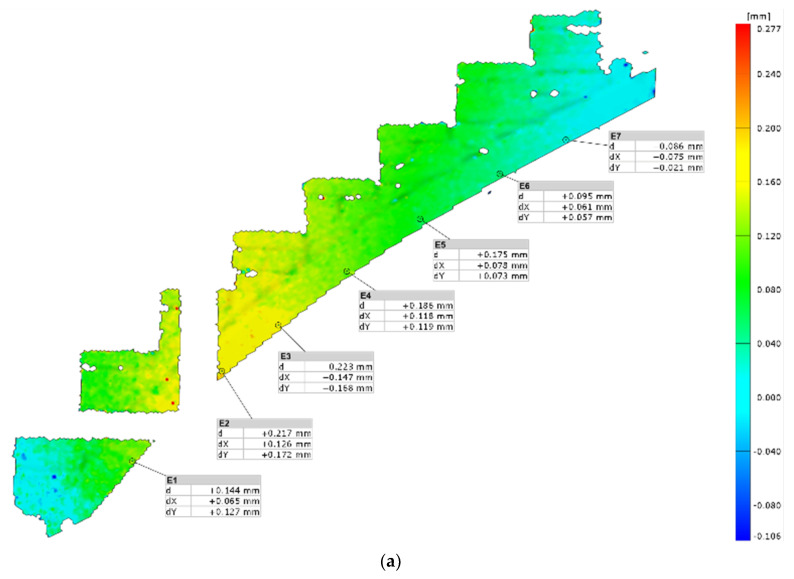

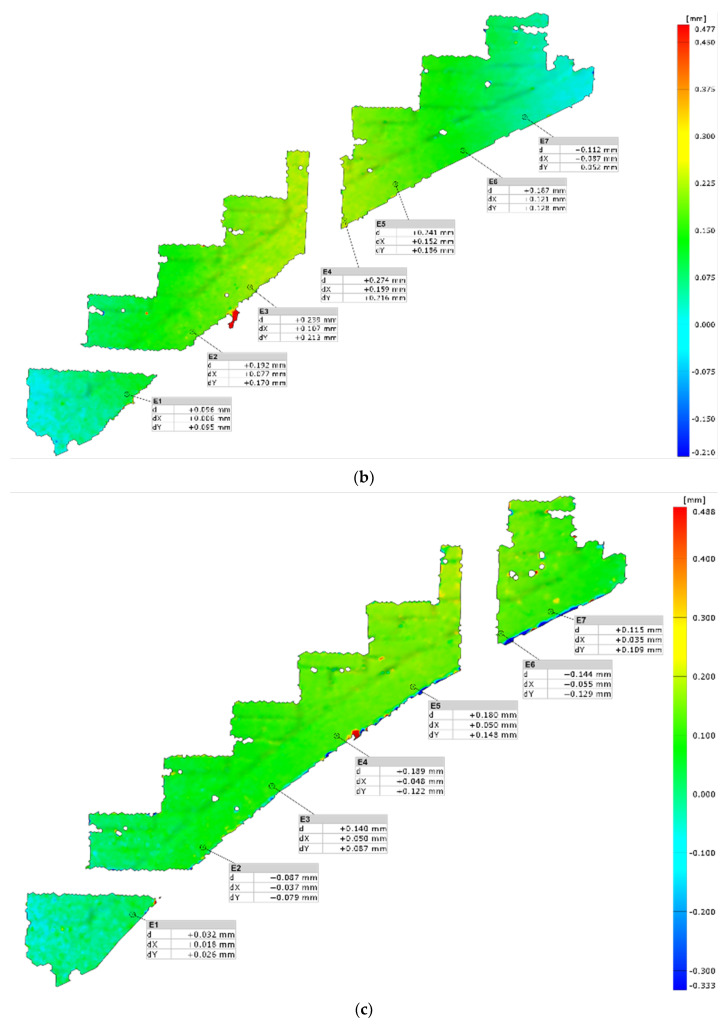

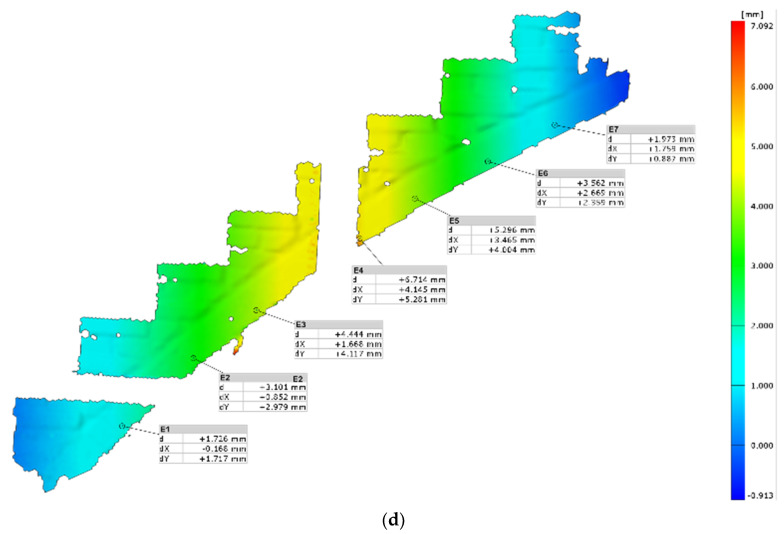
Results of experimental test of masonry staircases—displacement measured by ARAMIS: load P = 6 kN at point P_1_ (bottom) (**a**), load P = 6 kN at point P_2_ (middle) (**b**), load P = 6 kN at point P_3_ (top) (**c**) and load at failure P = 59.8 kN in point P_2_ (**d**). d—resultant displacement; dX—horizontal displacement; dY—vertical displacement.

The results measured using the ARAMIS system allowed us to analyze the displacement with higher precision. For the reference load level P = 6 kN, there were visible differences in the operation of the structure depending on the load application point. Analyzing the displacement maps (Figure 12), it is visible that there were local deformations. Those were caused by the movement of the bricks toward each other due to the changes in the joints.

For model M2, regardless of the load application point, the values of arch displacement were of the same sign. This is different from a typical operation of a masonry arch. The biggest displacement of the staircase was achieved when the force was applied at point P_2_. The results for points P_1_ (value for E4, virtual point, in the middle, d = 0.186 mm) and P_3_ (also value for E4, virtual point, in the middle, d = 0.189 mm) were similar. The values of deflections at the points directly under the applied forces differed for P_1_ (virtual point E2, d = 0.217 mm) and P_3_ (virtual point E6, d = 0.144 mm), even though the total deflection at point E2 was similar. For the load level P = 6 kN, the highest resultant deflection for point P_2_ was d = 0.274 mm. This is a value that can be hardly seen with a naked eye. The failure of the structure was also at a small resultant displacement of d = 6.714 mm. These results confirm a significant influence of masonry treads on the rigidity of the whole structure. The treads are responsible for increased stiffness of a lean masonry arch. Due to the low values of the displacement of treads, the diagnostic of the structure should be performed with precise measurement equipment, such as 3D scanners (TLS).

## 4. Conclusions

Based on the research study presented, the following conclusions were drawn:

- The TLS method allowed us to perform the geometric analysis and dimensioning of the existing arched stair structure.
- The TLS measurements allowed us to detect geometric irregularities, which, complemented by visual diagnostic tests, allowed us to detect damage to their structure.
- The damage to the flight was caused by improperly performed repair works—improper temporary support of the flight.
- The conducted experimental investigations showed that the key element responsible for the actual load-bearing capacity and stiffness of the stair flight were the treads above the masonry arch.
- The load-bearing capacity of the model with treads was 13.3 times higher than that of the model without treads.
- The deflection value of model M2 was 9.5 times smaller than the deflection of model M1.
- A different working mechanism was found for the stair arch model with brick threads (M2) compared to the arch without threads (M1).
- The failure of model M1 was caused by the opening of the crack within the joint located under the concentrated force at point P_2_.
- In the case of model M2, failure occurred as a result of cracking along the arch, at the interface of the arch with the brick treads.
- In the case of arched stair renovation, it is crucial to properly connect the arches of the stair flights with the treads above. If there is no proper connection between the vaults and the treads, the increase in the load-bearing capacity shown in the research study should be excluded from the calculations.

## Figures and Tables

**Table 1:** Ultrasonic pulse velocity and calculated compressive strength of tested bricks and mortar.

Ordinal No	Bricks		Mortar	
	Ultrasonic Pulse Velocity (m/s)	Compressive Strength (MPa)	Ultrasonic Pulse Velocity (m/s)	Compressive Strength (MPa)
**1**	1130	14.3	1192	3.6
**2**	971	10.4	1227	3.9
**3**	1472	28.4	1317	4.6
**4**	1172	15.6	1414	5.3
**5**	1071	12.7	1276	4.3
**6**	1327	21.2	994	2.1
**7**	1191	16.2	1321	4.6
**8**	1432	26.2	1417	5.4
**9**	1101	13.5	1276	4.3
**Mean value**	1207.4	17.6	1270.4	4.2

**Table 2:** Properties of used materials.

Load Diagram	Test	Material	No. of Samples	Result	Coefficient of Variation
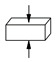	Compressive strength [108]	Brick	6	*f_b_* = 26.6 MPa	9%
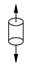	Tensile strength	Brick	6	*f_tb_* = 2.3 MPa	18%
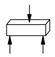	Flexural strength [109]	Mortar	9	*f* = 2.7 MPa	17%
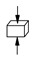	Compressive strength [109]	Mortar	12	*f_m_* = 8.0 MPa	23%
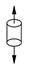	Tensile strength	Mortar	6	*f_tm_* = 1.0 MPa	13%
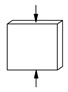	Compressive strength [110] Young’s modulus [110] Poisson’s coefficient [110]	Masonry	6	*f_m_* = 10.8 MPa *E* = 6.6 GPa *ν* = 0.14	7% 7% 17%
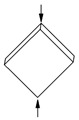	Tensile splitting strength [111]	Masonry	6	*f_t_*_45_ = 0.52 MPa	11%
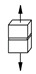	Tensile strength	Masonry	7	*f_t_*_90_ = 0.11 MPa	1%
